# A 10-Year Bibliometric Analysis of Global Research on Gut Microbiota and Parkinson's Disease: Characteristics, Impact, and Trends

**DOI:** 10.1155/2022/4144781

**Published:** 2022-06-27

**Authors:** Miguel Cabanillas-Lazo, Carlos Quispe-Vicuña, John Barja-Ore, Alicia Fernandez-Giusti, Arnaldo Munive-Degregori, Yesenia Retamozo-Siancas, Maria Eugenia Guerrero, Frank Mayta-Tovalino

**Affiliations:** ^1^Sociedad Científica San Fernando, Universidad Nacional Mayor de San Marcos, Lima, Peru; ^2^Grupo Peruano de Investigación Epidemiológica, Unidad para la Generación y Síntesis de Evidencias en Salud, Universidad San Ignacio de Loyola, Lima, Peru; ^3^Dirección de Investigación, Universidad Privada del Norte, Lima, Peru; ^4^Postgradute Department, Faculty of Medicine, Universidad Nacional Mayor de San Marcos, Lima, Peru; ^5^Master's Program in Library and Information Sciences, Faculty of Letters and Human Sciences, Universidad Nacional Mayor de San Marcos, Lima, Peru; ^6^Academic Department of Medical and Surgical Stomatology, Universidad Nacional Mayor de San Marcos, Lima, Peru; ^7^Vicerrectorado de Investigación, Universidad San Ignacio de Loyola, Lima, Peru

## Abstract

**Objective:**

To perform a bibliometric analysis of scientific production related to gut microbiota and Parkinson's disease between 2011 and 2020.

**Methods:**

A descriptive, retrospective, cross-sectional, and bibliometric study was carried out. The Scopus database was used as a source to evaluate the worldwide scientific production on intestinal microbiota and its relationship with Parkinson's disease. Data were extracted from Scopus using a formula developed with thesaurus terms MeSH (Medline) and Emtree (Embase).

**Results:**

A total of 591 documents were found. The retrieved manuscripts received an average of 41.9 citations per document. Four of the 10 most productive authors were Italian. The University of Helsinki (Finland) was the institution with the highest scientific production (19 papers) and the highest impact (5921 citations). In terms of productivity and impact, Movement Disorders ranked first with 38 papers and 2782 citations, and those papers published in Q1 quartile journals exceeded the sum of the remaining quartiles. Papers with international collaboration were the most cited. Keyword analysis showed that the terms Parkinson Disease, Disease, and Intestine Flora were the most frequent.

**Conclusion:**

The number of papers on Parkinson's disease and gut microbiota has been increasing; however, high-quality journals maintain the same high publication rate. International collaboration from high-income countries played an important role in the impact generated by the publications.

## 1. Introduction

Parkinson's disease (PD) is a common neurodegenerative disorder that is age-related. It has an approximate prevalence of more than 6 million people worldwide [1]. Due to the increasing longevity of the world population, the average number of people with PD is predicted to increase by 2030. However, a correct understanding of the pathophysiology and clinical manifestation of this disease is still uncertain [2].

PD is expressed by a decrease in dopamine levels which in turn triggers motor disturbances such as tremor, loss of balance, and rigidity [3]. It has been reported that 80 to 90% of these patients have gastrointestinal symptoms such as constipation [4, 5], mainly due to degeneration of dopaminergic cells of the enteric system [6]. Due to the initial involvement of the gastrointestinal tract and even before the first motor symptoms occur [7], the “gut-brain axis” has been considered where the composition of the gut microbiota has been shown to play an important role and is therefore being investigated with increasing impetus in the fields investigating biological and physiological bases of neurodegenerative and psychiatric diseases [8].

In addition to the report of intestinal dysbiosis present in PD patients [9, 10], the relationship between the alteration of specific bacterial species with the clinical features of PD has been reported [11]. This close relationship has led in recent years to clinical trials aimed at modifying the microbiota in PD patients to alleviate symptoms and complications [12–14]. Although the scientific evidence is extensive worldwide, it has not been systematically analyzed by bibliometric analysis.

Bibliometrics is the use of different statistical methods to analyze scientific publications through different indicators of production, impact, and collaboration. In addition, authors, institutions, keywords, etc. are evaluated. Bibliometric analyses have been used to explore the scientific production of researchers, institutions, and regions in certain areas [15–17]. This is to measure the quality of educational and research programs of an institution to elaborate strategic objectives [18]. In addition, it has been used to identify the influence of citations between journals [19] in relation to scientific growth, to investigate the collaborative structure in an interdisciplinary field [20], and to identify the thematic structure on a topic [21]. Previous bibliometric manuscripts have focused on the gut microbiome in depression [22], obesity [23], and the “microbiota-gut-brain” axis [24]. Our analysis covers the relationship between gut microbiota issues and Parkinson's disease. Our results could be useful for researchers developing their studies in this field so that they can identify potential related journals, collaborators, and institutions.

Therefore, the aim of this research was to analyze the status and characteristics of current trends in worldwide publications on gut microbiota and Parkinson's disease through a bibliometric analysis.

## 2. Methods

### 2.1. Database

The Scopus database (Elsevier BV Company, United States, available at: https://www.scopus.com/) was used to access the metadata of the manuscripts. It was decided to work with this database because of its scope since it condenses international data and has a wide range of scientific journals compared to other databases [25]. In addition, the SciVal tool was used to perform the bibliometric analysis due to its compatibility with Scopus since both are supported by Elsevier.

The following selection criteria were used: original article, reviews, and any language on the subject published in the period 2011-2020. On the other hand, letters to the editor, notes, proceedings, and publications not listed in Scopus were excluded.

Finally, this study only worked with manuscripts published in Scopus because SciVal is a tool created by Elsevier; so, it is compatible to perform bibliometric studies since the data and software belong to the same makers of Elsevier.

### 2.2. Search Strategy

The MeSH and Emtree terms from PubMed and Embase were used, respectively. The “AND” and “OR” operators were used to elaborate the final search strategy. In addition, the truncator (^∗^) was used to increase the scope of the search for words that share the same root. The search was performed in Medicine, and a cut-off period of 2011-2020 was established in SciVal.

The following strategy was established: TITLE-ABS-KEY (“Idiopathic Parkinson's Disease” OR “Lewy Body Parkinson's Disease” OR “Parkinson's Disease, Idiopathic” OR “Parkinson's Disease, Lewy Body” OR “Parkinson Disease, Idiopathic” OR “Parkinson's Disease^∗^” OR “Idiopathic Parkinson Disease” OR “Lewy Bod^∗^ Parkinson^∗^ Disease^∗^” OR “Primary Parkinsonism” OR “Parkinsonism, Primary” OR “Paralysis Agitans” OR “idiopathic parkinsonism” OR “Parkinson dementia complex”) AND TITLE-ABS-KEY (“lactobacill^∗^” OR “bifidobacter^∗^” OR “enterococ^∗^” OR “saccharom^∗^” OR “streptoc^∗^” OR “escheric^∗^” OR “probiot^∗^” OR “Prebiot^∗^” OR “Dietary Fiber^∗^” OR “Wheat Bran^∗^” OR “Roughage^∗^” OR “Dietary Carbohydrate^∗^” OR “Synbiot^∗^” OR “dysbios^∗^” OR “gut intestine^∗^ flora” OR “microbiota^∗^” OR “microbiome^∗^” OR “flora” OR “gut microflora”) AND (LIMIT-TO (PUBSTAGE, “final”)).

### 2.3. Data Analysis

On September 19, 2021, the data corresponding to the period 2011-2020 were downloaded and exported from Scopus in .csv format. This was since the year 2021 was not yet in force in SciVal because it was still a current year. They were then analyzed with the SciVal tool (Elsevier BV Company, USA, available at https://www.scival.com/), where some indicators of production, impact, and collaboration were identified.

The following bibliometric indicators were established: (a) number of papers and most productive journals, universities, and authors publishing scientific papers on Parkinson's disease and gut microbiota; (b) collaboration in research related to the topic; (c) citation count; (d) document count; (e) citations per document; (f) CiteScore calculates the average number of citations received in a calendar year for all articles published in that journal in the previous 3 years [26]; and (g) Scimago Journal and Rank weights the value of a citation based on the field, quality, and reputation of the journal from which the citation originates [26].

The VOSviewer software (version 1.6.10) was used to analyze the most important collaborative networks [27]. In addition, a thesaurus was elaborated to merge singular and plural words.

## 3. Results

A total of 591 documents were retrieved from the Medicine Category, with 24750 citations, 3243 citations and, in addition, an average of 41.9 citations per document. Most of the retrieved documents were published in the following subcategories: neurology (*n* = 219; 37.1%), general medicine (*n* = 61; 10.3%), pharmacology (*n* = 47; 8.0%), gastroenterology (*n* = 45; 7.6%), and immunology and allergy (*n* = 44; 7.4%).

### 3.1. Top Ten Most Productive Authors

Table 1 shows the authors with the highest production in gut microbiota and Parkinson's disease. Scheperjans with affiliation from Helsinki University Hospital in Finland leads the list with the highest number of papers (12) and with the highest number of citations (1122) followed in citations by Cryan with 1088.

### 3.2. Top Ten Most Productive Institutions

The top 10 universities with the highest number of papers are shown in Table 2. The University of Helsinki (Finland) was the institution with the highest scientific production (19) and the highest impact for having the highest number of citations (5921). The Institut National de la Santé et de la Recherche Médicale (France) and Harvard University (USA) were the second and third institutions with the highest scientific production, respectively.

### 3.3. Top Ten Most Productive Journals

The top 10 journals with the highest number of publications in gut microbiota and Parkinson's disease are shown in Table 3. The first three places went to Movement Disorders, Parkinsonism and Related Disorders, and Journal of Parkinson's Disease with 38, 19, and 19 papers, respectively. However, only the first two maintain their place in terms of the highest citation (2782 and 1047, respectively). Nature Reviews Neurology obtained a significant citations/paper.

In addition, according to the CiteScore, Table 4 shows the number of papers according to the quartile of the journal between 2011 and 2020. The high concentration of these publications in Q1 suggests the high quality. It should be noted that, as of 2013, publications in journals in the first quartile exceed the sum of the remaining quartiles.

### 3.4. Document Collaboration Networks

Table 5 shows the type of collaboration and its bibliometric indicators. Most of the retrieved papers had only national collaboration (*n* = 188; 31.9%), followed by only institutional collaboration (*n* = 184; 31.2%) and international collaboration (*n* = 126; 21.4%). However, in terms of impact, international collaboration (11249; 89.3 citations/paper) exceeds both national (6747; 35.9) and institutional (5433; 29.5). The rest of the documents belong to the “single authorship” or “no collaboration” category (*n* = 91; 15.4%).

### 3.5. Visualization of the Document Network

**Figure 1:**
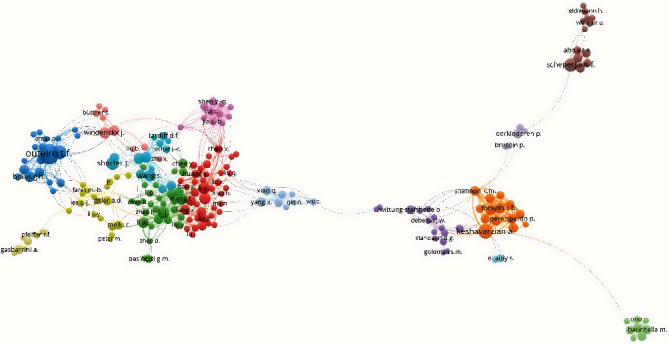
Collaborative scientific networks between authors.

**Figure 2:**
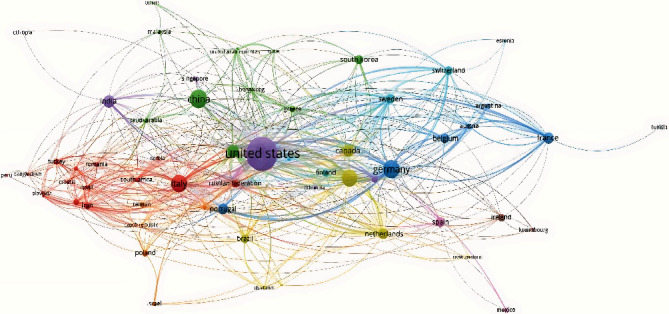
Crosscountry scientific collaboration networks.

**Figure 3:**
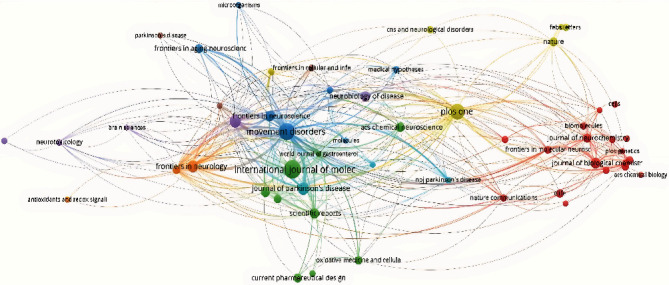
Bibliometric map of cocited journals.

**Figure 4:**
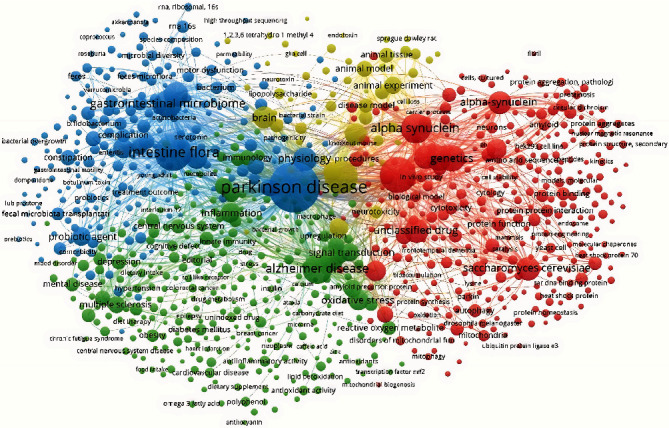
Research topics clustered by mapping co-occurrences of terms.

Figure 1 shows the collaboration network among authors with more than 3 papers.  Figure 2 shows the collaboration network between countries with at least 2 papers. The United States is the country with the highest occurrence of coauthorships.  Figure 3 shows the collaborative network among authors with more than 3 papers. Movement Disorders and International Journal of Molecular Sciences are the most cocited journals.  Figure 4 shows the co-occurrences of terms at least 10 times. “Parkinson Disease” and “Intestine Flora” are the most frequent terms.

## 4. Discussion

In recent years, an association between microbiota and neurological diseases has been found. Recent studies have demonstrated alterations in the intestinal microbiota in PD patients as well as a possible use of this microbiota as a therapeutic target, so that in the coming years the number of publications will increase significantly. The present research constitutes the first study that analyzes the worldwide scientific production on PD and gut microbiota, with emphasis on the category Medicine. This research assumes that the impact of a study is expressed in the citations it receives from the world literature once it is published.

Bibliometric analyses are used to evaluate the characteristics of published scientific research, especially in specialized scientific fields [28]. Scopus is an extensive database and has tools for citation and author description. In addition, Scopus has many documents and references compared to other databases such as Web of Science; so, it provides a broader perspective [29]. That is why this database has already been used in other bibliometric studies referring to the gut microbiome [24, 30].

When evaluating the data for the period 2011-2020, Scheperjans (Finland) is the author with the largest number of papers and is the most influential in gut microbiota and PD research by far with respect to the other authors. One of his greatest contributions is the suggestion that the gut microbiome is altered in PD and is associated with the motor phenotype of PD such as postural instability and difficulty in walking [31]. On the other hand, in the same period, the author with the most citations per paper was Cryan. This may be because the author published narrative reviews in first quartile journals that were cited in large numbers. In addition, it is worth noting that four of the top 10 authors with the highest output are Italian. This may be explained by the large increase in Parkinson-related articles from this country, which was reported in a bibliometric study conducted by Li et al. [32].

In terms of the 10 most prolific institutions, U.S. institutions were the most productive. University of Helsinki was the institution with the highest production and impact. It is also important to note that University College London is the institution with the highest citation per paper. This is consistent with a bibliometric study by Shafiei et al. who reported that this institution is the most productive in the field of movement disorders; so, this denotes intrainstitutional collaboration and a line of research directed to be at the forefront of global research in this field [33].

Movement Disorders was the journal with the highest number of papers (38) and citations (2782). In addition, it has remained among the most productive journals among other bibliometric studies, ranking first in Deep Brain Stimulation Treatment for Dystonia [34] and in the field of movement disorders [33]. In addition, it should be noted that more than half of the papers were published in first quartile journals, and this percentage has been sustained over time (2011-2020). This would demonstrate great interest on the part of the scientific community in this topic.

In our analysis, national collaborative papers predominated over international ones. These results are like those of a bibliometric analysis on multiple sclerosis [35]. In addition, it is noteworthy that the number of citations of papers with international collaboration was the highest. The latter is consistent with a bibliometric analysis on palliative care in South America [36] and another on scientific output in Eastern European academic institutions [37]. International collaboration is necessary to address necessities, generate new and important publications, and exchange opportunities and relevant information [38].

Finally, our research has some limitations. First, only the last 10 years were analyzed; so, some studies on the subject were excluded, representing more than 80% of all available papers on the topic in Scopus. Second, like other bibliometric studies, some papers may have been omitted because they were published in journals not indexed in Scopus. However, this is the first study on Parkinson's disease and gut microbiota applying bibliometric indicators. Third, we only analyzed bibliometric information from the Scopus database, which does not reflect the totality of publications on the subject. However, Scopus only includes journals that met a strict peer review process and high standards [29].

## 5. Conclusions

The number of papers on Parkinson's disease and gut microbiota has been increasing; however, high-quality journals have maintained the same high publication rate over the last decade. International collaboration from high-income countries plays an important role in the impact generated by publications. Joint efforts between institutions and researchers from different countries are needed to establish connections and future research to expand knowledge on this growing and novel topic.

## Figures and Tables

**Table 1:** Top ten authors publishing on gut microbiota and Parkinson disease.

Author	Documents, *n* (%)	Total citation	Citations per document	*h*-index	FWCI	Country
Scheperjans, Filip	12 (2.0)	1122	93.5	18	5.5	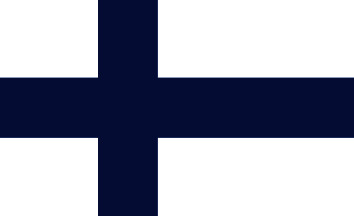
Unger, Marcus Michael	7 (1.2)	505	72.1	20	4.9	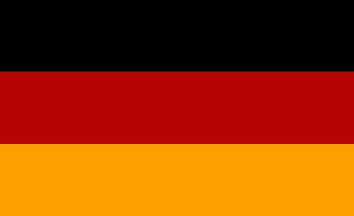
Derkinderen, Pascal	7 (1.2)	249	35.6	44	2.4	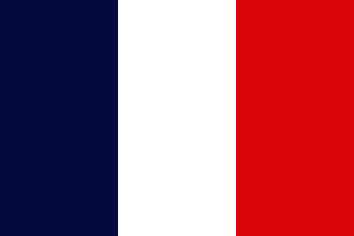
Keshavarzian, Ali	7 (1.2)	669	95.6	70	5.4	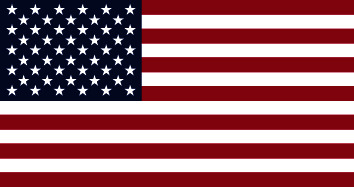
Faßbender, Klaus C.	6 (1.0)	505	84.2	53	5.8	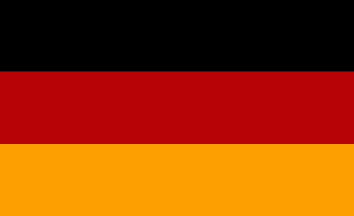
Cassani, Erica	6 (1.0)	352	58.7	18	3.9	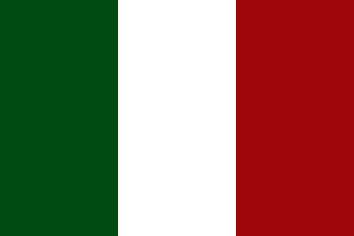
Gasbarrini, Antonio	6 (1.0)	750	125	82	13.00	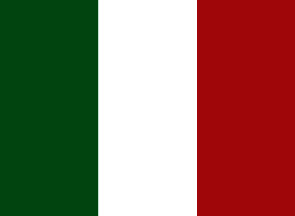
Barichella, Michela	6 (1.0)	352	58.7	26	3.9	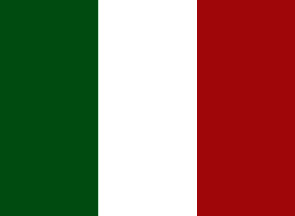
Cryan, John F.	6 (1.0)	1088	181.3	109	18.4	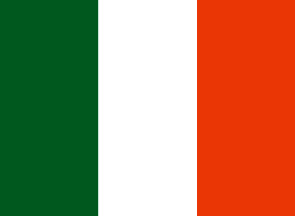
Pezzoli, Gianni	6 (1.0)	352	58.7	62	3.9	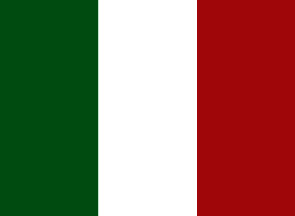

FWCI: field-weighted citation impact.

**Table 2:** Top ten productive institutions on gut microbiota and Parkinson disease.

Institution (country)	Documents, *n* (%)	Total citation	Authors	Citations per document	FWCI
University of Helsinki (Finland)	19 (3.2)	5921	23	311.6	35.4
Institut National de la Santé et de la Recherche Médicale (France)	16 (2.7)	413	49	25.8	2.1
Harvard University (United States)	15 (2.5)	5245	103	349.7	43.3
King's College London (United Kingdom)	13 (2.2)	4903	32	377.2	47.9
Rush University (United States)	13 (2.2)	969	28	74.5	4.4
University of Toronto (Canada)	12 (2.0)	5365	43	447.1	55.4
Department of Veterans Affairs (United States)	12 (2.0)	5323	25	443.6	53.9
VA Medical Center (United States)	11 (1.9)	4954	26	450.4	56.8
University of Groningen (Netherlands)	10 (1.7)	4671	33	467.1	60.5
University College London (United Kingdom)	9 (1.5)	4825	31	536.1	67.3

FWCI: field-weighted citation impact.

**Table 3:** Bibliometric indicators of production and impact on journals on gut microbiota and Parkinson disease.

Journals	Quartile	Scimago Journal Rank	Documents	Citations	Citations per document	CiteScore 2020
Movement Disorders	Q1	3.4	38	2782	73.2	13.3
Parkinsonism and Related Disorders	Q1	1.5	19	1047	55.1	6.2
Journal of Parkinson's Disease	Q1	1.7	19	304	16	6.8
Frontiers in Neurology	Q2	1.2	18	330	18.3	4.0
Frontiers in Immunology	Q1	2.6	12	379	31.6	8.1
Prion	Q3	0.6	9	227	25.2	3.0
Medical Hypotheses	Q3	0.4	7	108	15.4	2.4
Human Molecular Genetics	Q1	2.8	7	374	53.4	9.6
Frontiers in Cellular and Infection Microbiology	Q1	1.8	7	103	14.7	6.5
Nature Reviews Neurology	Q1	7.3	6	429	71.5	29.5

**Table 4:** Documents published according to CiteScore Quartile 2020 on gut microbiota and Parkinson disease (2011–2020).

CiteScore Quartile	2011	2012	2013	2014	2015	2016	2017	2018	2019	2020	Total
Q1	5	9	15	16	23	24	38	48	56	74	308
Q2	5	6	6	6	10	10	9	18	23	33	126
Q3	7	2	3	2	4	5	2	12	15	17	69
Q4	2	1	1	1	2	6	6	9	14	9	51
Total	19	18	25	25	39	45	55	87	108	133	554

**Table 5:** Bibliometric indicators of production and impact according to type of collaboration on gut microbiota and Parkinson disease.

Collaboration	%	Documents	Citations	Citations per document	FWCI
International	21.4	126	11249	89.3	8.5
Only national	31.9	188	6747	35.9	3.0
Only institutional	31.2	184	5433	29.5	2.3
Single authorship (no collaboration)	15.4	91	1318	14.5	1.4

FWCI: field-weighted citation impact.

## Data Availability

The data used in the statistical analysis of this study will be available upon authorization of the corresponding author.
